# Supplementary material for: Accumulation of sequence variants in genes of Wnt signaling and focal adhesion pathways in human corneas further explains their involvement in keratoconus
Source: PeerJ. 2020 Apr 14;8:e8982. doi: 10.7717/peerj.8982 (PMC7164425; doi:10.7717/peerj.8982)
Supplement: Supplemental Information 1 — aPK—penetrating keratoplasty bOD—Right eye cOS—Left eye [file peerj-08-8982-s001.docx]

**Supplementary Table 1**. The clinical characteristics of examined individuals

| **Id** | **Gender** | **Age at diagnosis** | **Age at surgery** | **Type of surgery (eye)** | **diff BFS [µm]** | | **Min. Pachymetry [µm]** | | **Axial length [mm]** | | **Eye rubbing** |
| --- | --- | --- | --- | --- | --- | --- | --- | --- | --- | --- | --- |
|  |  |  |  |  | **OD**^b^ | **OS**^c^ | **OD** | **OS** | **OD** | **OS** |  |
| KC15 | F | 21 | 46 | PK^a^ (OS) | NA | NA | 164 | 303 | 22.73 | 21.72 | Yes |
| KC16 | M | 21 | 30 | PK (OS) | NA | NA | 303 | 314 | 24.62 | 23.74 | No |
| KC17 | F | 20 | 46 | PK (OS) | 204 | NA | 272 | NA | 24.20 | 22.43 | No |
| KC18 | M | 24 | 35 | PK (OS) | 68 | 152 | 444 | 366 | 24.19 | 23.82 | No |
| KC19 | M | 30 | 51 | PK (OS) | 92 | 254 | 446 | 359 | 27.60 | 27.35 | No |

^a^PK – penetrating keratoplasty

^b^OD – Right eye

^c^OS – Left eye
